# *catena*-Poly[[tetra-μ-formato-κ^8^ *O*:*O*′-dicopper(II)]-μ-hexa­methyl­ene­tetra­mine-κ^2^ *N* ^1^:*N* ^5^]

**DOI:** 10.1107/S160053681303184X

**Published:** 2013-11-30

**Authors:** Jianfang Cao, Ziping Huang, Changnian Cao, Chunchun Cheng, Chunyan Sun

**Affiliations:** a Chemical Engineering College, Qinghai University, Xining 810016, People’s Republic of China

## Abstract

In the title polymeric compound, [Cu_2_(HCO_2_)_4_(C_6_H_12_N_4_)]_*n*_, the Cu^II^ atom is five-coordinated in a square-pyramidal geometry that is defined by four O atoms from four formate ligands and one N atom from a hexa­methyl­ene­tetra­mine ligand. The two Cu^II^ atoms are separated by 2.6850 (7) Å, and together with the four formate ligands they form a paddle-wheel unit. The hexa­mine ligand uses only two of its four N atoms to link Cu_2_ cluster units, affording a zigzag chain running along the *b-*axis direction. The hexa­mine ligand lies on a mirror plane.

## Related literature

For background to hexa­mine chemistry, see: Dreyfors *et al.* (1989 ▶); Kirillov (2011 ▶). For hexa­mine as a bridging ligand, see: Pickardt (1981 ▶); Konar *et al.* (2003 ▶); Wang *et al.* (2002 ▶). For paddle-wheel Cu_2_-cluster units, see: Konar *et al.* (2003 ▶); Chiari *et al.* (1988 ▶); Wu & Wang (2004 ▶); Sun *et al.* (2009 ▶).
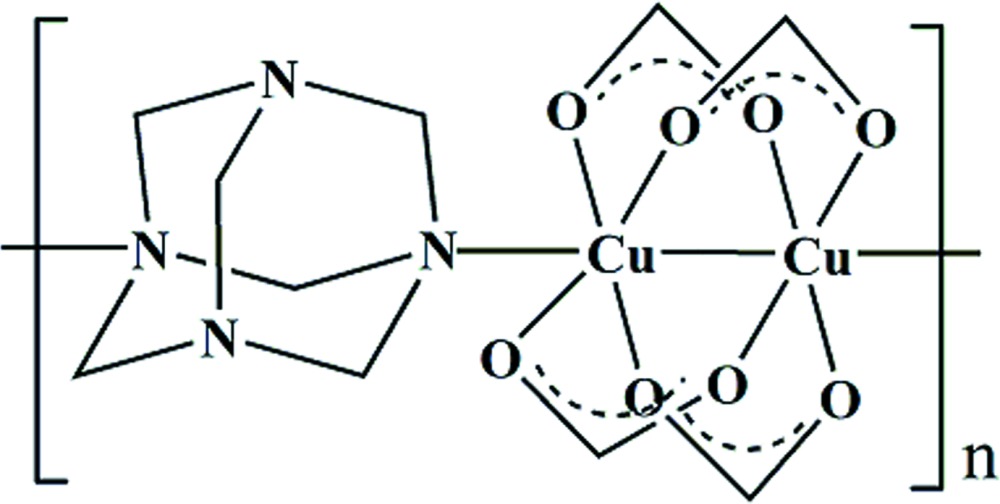


## Experimental

### 

#### Crystal data

[Cu_2_(CHO_2_)_4_(C_6_H_12_N_4_)]
*M* *_r_* = 447.36
Orthorhombic, 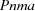

*a* = 13.1252 (19) Å
*b* = 17.281 (3) Å
*c* = 6.4777 (9) Å
*V* = 1469.3 (4) Å^3^
*Z* = 4
Mo *K*α radiation
μ = 2.95 mm^−1^
*T* = 103 K
0.26 × 0.24 × 0.18 mm

#### Data collection

Bruker SMART APEX area-detector diffractometer
Absorption correction: multi-scan (*SADABS*; Sheldrick, 1996 ▶) *T*_min_ = 0.469, *T*_max_ = 0.588
5203 measured reflections
1550 independent reflections
1345 reflections with *I* > 2σ(*I*)
*R*_int_ = 0.021

#### Refinement

*R*[*F* ^2^ > 2σ(*F* ^2^)] = 0.028
*wR*(*F* ^2^) = 0.077
*S* = 1.04
1550 reflections
115 parameters
H-atom parameters constrained
Δρ_max_ = 0.69 e Å^−3^
Δρ_min_ = −0.71 e Å^−3^

### 

Data collection: *SMART* (Bruker, 1997 ▶); cell refinement: *SAINT* (Bruker, 1997 ▶); data reduction: *SAINT*; program(s) used to solve structure: *SHELXS97* (Sheldrick, 2008 ▶); program(s) used to refine structure: *SHELXL97* (Sheldrick, 2008 ▶); molecular graphics: *SHELXTL* (Sheldrick, 2008 ▶); software used to prepare material for publication: *SHELXTL*.

## Supplementary Material

Crystal structure: contains datablock(s) I. DOI: 10.1107/S160053681303184X/ng5345sup1.cif

Structure factors: contains datablock(s) I. DOI: 10.1107/S160053681303184X/ng5345Isup2.hkl

Supplementary material file. DOI: 10.1107/S160053681303184X/ng5345Isup3.mol

Additional supplementary materials:  crystallographic information; 3D view; checkCIF report

## Figures and Tables

**Fig. 1.**
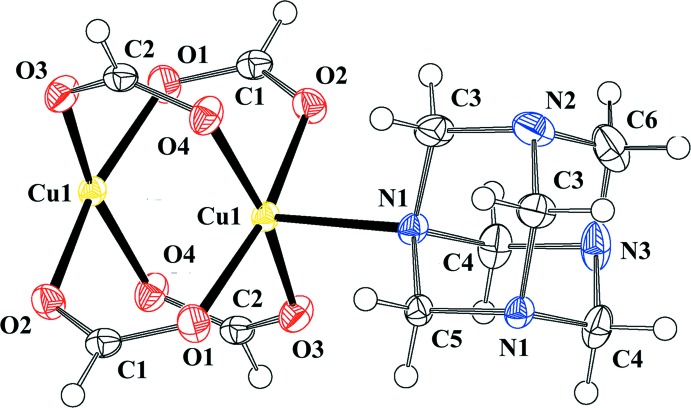
The molecular structure of [Cu2(HCO2)4(C6H12N4)]n showing the atomic dlabelling scheme. Displacement ellipsoids are drawn at the 30% probability level.

**Fig. 2.**
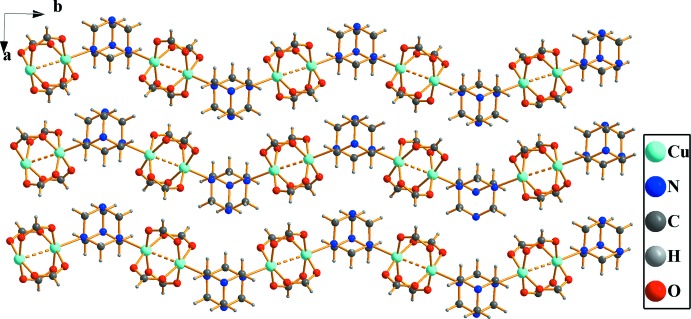
The zigzag chain in the crystal packing structure of [Cu2(HCO2)4(C6H12N4)]n along b the axis.

## supplementary crystallographic information

### 1. Comment

The design and synthesis of metal-organic complexes or coordination polymers is a rapidly developing field in coordination and supramolecular chemistry during the past decades. Hexamethylenetetramine (hmt), also known as hexamine or urotropine, can be considered as one such simple heterocyclic compound with a cagelike structure which,owing to its high solubility in water and polar organic solvents, has found a broad variety of applications (Dreyfors *et al.*, 1989). With regard to coordination chemistry, hmt is a versatile ligand capable of adopting different coordination modes that span from the terminal monodentate to bridging bi-, tri- and tetradentate modes. The well known [Cu_2_(carboxylate)_4_] units with four bridging carboxylate ligands in the familiar *η*_1_:*η*_1_:*µ* coordination mode have accessible apical coordination sites and are ideally suited to serve as a metal-based linear spacer (Konar *et al.* 2003; Chiari *et al.*, 1988; Wu *et al.*, 2004; Sun *et al.*, 2009). To date, the title copper(II) carboxylate complex, represents an exception, as only few formate copper(II) complexes have been studied.

The crystal structure of the title complex, which is isostructural with its copper analog (Wang *et al.*, 2002), is built of hexamethylenetetramine molecules and paddle-wheel dicopper units, both of which occupy special positions. The structure of the centrosymmetric [Cu_2_(HCO_2_)_4_] moiety is shown in Fig. 1. The coordination geometry of the Cu atom may be described as a square pyramid, formed by four formatee O atoms and the N atom of the hexamine . The four basal Cu—O distances fall in the range from 1.963 (2) to 1.978 (2) Å. The central hmt has mirror symmetry, and therefore there is only one independent Cu^II^ atom in the asymmetric unit. The Cu—Cu distance within the [Cu_2_(HCO_2_)_4_] unit is 2.6850 (7) Å indicating a strong interaction. The axial Cu(1)—N(1) distance is 2.212 (2) Å. The hexamine ligand uses only two of its four N atoms to link adjacent paddle-wheel Cu_2_-cluster units, to afford a zigzag chain running along the *b* axis of the unit cell (Fig. 2).

### 2. Experimental

The title compound was synthesized by the following method. Copper(II) formate tetrahydrate (0.015 g, 0.1 mmol) was dissolved in 20 ml me thanol to obtain solution A. Hexamine (0.007 g, 0.05 mmol) was dissolved in 10 ml methanol to obtain solution B. Solution B was layered carefully on solution A, and the tube was sealed and stored in room temperature. Green block crystals were obtained after two weeks. Analysis calculated for C_5_H_8_CuN_2_O_4_: C 26.85, H 3.60, N 12.52%; found: C 26.66, H 3.82, N 12.65%.

### 3. Refinement

All non-hydrogen atoms were refined anisotropically. The H atoms of formate were positioned geometrically and allowed to ride on their parent atoms, with C–H = 0.95 Å and *U*_iso_(H) = 1.2*U*_eq_(C). The H atoms of hexamethylenetetramine the were placed in geometrically idealized positions and refined as riding atoms, with C–H(CH_2_) = 0.99 Å and *U*_iso_(H) = 1.2*U*_eq_(C).

### Crystal data

**Table:**

[Cu_2_(CHO_2_)_4_(C_6_H_12_N_4_)]	*F*(000) = 904
*M**_r_* = 447.36	*D*_x_ = 2.022 Mg m^−^^3^*D*_m_ = 2.022 Mg m^−^^3^*D*_m_ measured by not measured
Orthorhombic, *P**n**m**a*	Mo *K*α radiation, λ = 0.71073 Å
Hall symbol: -P 2ac 2n	Cell parameters from 1680 reflections
*a* = 13.1252 (19) Å	θ = 2.4–26.4°
*b* = 17.281 (3) Å	µ = 2.95 mm^−^^1^
*c* = 6.4777 (9) Å	*T* = 103 K
*V* = 1469.3 (4) Å^3^	Block, green
*Z* = 4	0.26 × 0.24 × 0.18 mm

### Data collection

**Table:**

Bruker SMART APEX area-detector diffractometer	1550 independent reflections
Radiation source: fine-focus sealed tube	1345 reflections with *I* > 2σ(*I*)
Graphite monochromator	*R*_int_ = 0.021
Detector resolution: 16.0143 pixels mm^-1^	θ_max_ = 26.4°, θ_min_ = 2.4°
ω scans	*h* = −7→16
Absorption correction: multi-scan (*SADABS*; Sheldrick, 1996)	*k* = −16→21
*T*_min_ = 0.469, *T*_max_ = 0.588	*l* = −8→8
5203 measured reflections	

### Refinement

**Table:**

Refinement on *F*^2^	0 restraints
Least-squares matrix: full	H-atom parameters constrained
*R*[*F*^2^ > 2σ(*F*^2^)] = 0.028	*w* = 1/[σ^2^(*F*_o_^2^) + (0.0488*P*)^2^ + 0.5436*P*] where *P* = (*F*_o_^2^ + 2*F*_c_^2^)/3
*wR*(*F*^2^) = 0.077	(Δ/σ)_max_ = 0.001
*S* = 1.04	Δρ_max_ = 0.69 e Å^−^^3^
1550 reflections	Δρ_min_ = −0.71 e Å^−^^3^
115 parameters	

### Special details

**Table:**

Geometry. All e.s.d.'s (except the e.s.d. in the dihedral angle between two l.s. planes) are estimated using the full covariance matrix. The cell e.s.d.'s are taken into account individually in the estimation of e.s.d.'s in distances, angles and torsion angles; correlations between e.s.d.'s in cell parameters are only used when they are defined by crystal symmetry. An approximate (isotropic) treatment of cell e.s.d.'s is used for estimating e.s.d.'s involving l.s. planes.
Refinement. Refinement of *F*^2^ against ALL reflections. The weighted *R*-factor *wR* and goodness of fit *S* are based on *F*^2^, conventional *R*-factors *R* are based on *F*, with *F* set to zero for negative *F*^2^. The threshold expression of *F*^2^ > σ(*F*^2^) is used only for calculating *R*-factors(gt) *etc*. and is not relevant to the choice of reflections for refinement. *R*-factors based on *F*^2^ are statistically about twice as large as those based on *F*, and *R*-factors based on ALL data will be even larger.

### Fractional atomic coordinates and isotropic or equivalent isotropic displacement parameters (Å^2^)

**Table:**

	*x*	*y*	*z*	*U*_iso_*/*U*_eq_	Occ. (<1)
Cu1	0.03147 (2)	0.569254 (15)	0.06885 (4)	0.01482 (13)	
N1	0.09051 (15)	0.67876 (11)	0.1987 (3)	0.0167 (4)	
C6	0.2292 (3)	0.7500	0.4773 (7)	0.0376 (11)	
H6A	0.2607	0.7963	0.5407	0.045*	0.50
H6B	0.2607	0.7037	0.5407	0.045*	0.50
O1	−0.06061 (14)	0.61313 (10)	−0.1393 (3)	0.0243 (4)	
O2	−0.11320 (13)	0.49698 (10)	−0.2546 (3)	0.0222 (4)	
O3	−0.08446 (14)	0.55920 (9)	0.2587 (3)	0.0229 (4)	
O4	−0.13726 (14)	0.44265 (9)	0.1462 (3)	0.0228 (4)	
C1	−0.11161 (19)	0.56950 (13)	−0.2535 (4)	0.0189 (5)	
H1	−0.1544	0.5944	−0.3515	0.023*	
C2	−0.14152 (18)	0.50082 (13)	0.2619 (4)	0.0186 (5)	
H2	−0.1938	0.5007	0.3634	0.022*	
C3	0.20300 (19)	0.68165 (14)	0.1630 (4)	0.0256 (6)	
H3A	0.2347	0.6346	0.2222	0.031*	
H3B	0.2164	0.6817	0.0126	0.031*	
C4	0.0729 (2)	0.68132 (15)	0.4251 (4)	0.0256 (6)	
H4A	−0.0014	0.6813	0.4520	0.031*	
H4B	0.1020	0.6342	0.4889	0.031*	
C5	0.0455 (3)	0.7500	0.1069 (5)	0.0164 (7)	
H5A	−0.0290	0.7500	0.1301	0.020*	
H5B	0.0576	0.7500	−0.0440	0.020*	
N2	0.2502 (3)	0.7500	0.2553 (6)	0.0306 (8)	
N3	0.1187 (3)	0.7500	0.5219 (5)	0.0299 (8)	

### Atomic displacement parameters (Å^2^)

**Table:**

	*U*^11^	*U*^22^	*U*^33^	*U*^12^	*U*^13^	*U*^23^
Cu1	0.01667 (19)	0.01185 (19)	0.01595 (19)	−0.00041 (10)	−0.00034 (10)	−0.00042 (10)
N1	0.0205 (10)	0.0106 (9)	0.0191 (10)	0.0003 (8)	−0.0004 (8)	0.0002 (8)
C6	0.047 (3)	0.0180 (19)	0.048 (3)	0.000	−0.028 (2)	0.000
O1	0.0307 (10)	0.0168 (9)	0.0253 (9)	0.0018 (7)	−0.0092 (8)	0.0001 (7)
O2	0.0245 (9)	0.0181 (9)	0.0240 (9)	0.0009 (7)	−0.0066 (7)	0.0015 (7)
O3	0.0232 (10)	0.0207 (9)	0.0248 (10)	−0.0027 (7)	0.0062 (7)	−0.0043 (7)
O4	0.0253 (9)	0.0197 (9)	0.0234 (9)	−0.0044 (7)	0.0056 (8)	−0.0033 (7)
C1	0.0182 (12)	0.0205 (12)	0.0181 (13)	0.0038 (9)	0.0019 (9)	0.0032 (9)
C2	0.0171 (12)	0.0195 (12)	0.0192 (12)	0.0037 (10)	−0.0002 (9)	0.0018 (9)
C3	0.0215 (13)	0.0151 (12)	0.0401 (16)	0.0018 (10)	−0.0030 (11)	−0.0002 (11)
C4	0.0407 (16)	0.0161 (13)	0.0199 (13)	−0.0014 (12)	−0.0026 (11)	0.0029 (9)
C5	0.0196 (17)	0.0127 (16)	0.0169 (16)	0.000	−0.0021 (13)	0.000
N2	0.0226 (16)	0.0167 (15)	0.052 (2)	0.000	−0.0107 (14)	0.000
N3	0.053 (2)	0.0145 (15)	0.0219 (16)	0.000	−0.0136 (15)	0.000

### Geometric parameters (Å, º)

**Table:**

Cu1—O1	1.9632 (17)	O2—Cu1^i^	1.9769 (16)
Cu1—O3	1.9640 (18)	O3—C2	1.257 (3)
Cu1—O2^i^	1.9769 (17)	O4—C2	1.255 (3)
Cu1—O4^i^	1.9777 (18)	C2—H2	0.950
Cu1—N1	2.2112 (19)	O4—Cu1^i^	1.9777 (18)
Cu1—Cu1^i^	2.6848 (6)	C3—N2	1.461 (3)
N1—C4	1.485 (3)	C3—H3A	0.990
N1—C5	1.489 (3)	C3—H3B	0.990
N1—C3	1.495 (3)	C4—N3	1.471 (3)
C6—N2	1.464 (6)	C4—H4A	0.991
C6—N3	1.479 (6)	C4—H4B	0.990
C6—H6A	0.990	C5—N1^ii^	1.489 (3)
C6—H6B	0.990	C5—H5A	0.989
O1—C1	1.251 (3)	C5—H5B	0.990
O2—C1	1.253 (3)	N2—C3^ii^	1.461 (3)
C1—H1	0.951	N3—C4^ii^	1.471 (3)
			
O1—Cu1—O3	89.26 (8)	H5B—C5—N1	109.30
O1—Cu1—O2^i^	167.34 (7)	C4—N1—Cu1	110.27 (15)
H1—C1—O1	116.00	H4A—C4—N3	109.13
H1—C1—O2	116.04	H4B—C4—N3	109.16
O3—Cu1—O2^i^	89.33 (7)	C5—N1—Cu1	114.62 (15)
O1—Cu1—O4^i^	89.34 (8)	C3—N1—Cu1	108.41 (14)
O3—Cu1—O4^i^	167.36 (7)	N2—C6—N3	112.1 (3)
O2^i^—Cu1—O4^i^	89.28 (8)	C1—O1—Cu1	120.20 (15)
H2—C2—O3	116.33	C1—O2—Cu1^i^	124.51 (16)
H2—C2—O4	116.33	C2—O3—Cu1	122.90 (15)
O1—Cu1—N1	98.43 (7)	C2—O4—Cu1^i^	122.36 (16)
O3—Cu1—N1	96.26 (7)	H3A—C3—H3B	107.88
O2^i^—Cu1—N1	94.24 (7)	H4A—C4—H4B	107.83
O4^i^—Cu1—N1	96.37 (7)	H5B—C5—H5A	107.97
O1—Cu1—Cu1^i^	85.79 (5)	O1—C1—O2	127.9 (2)
O3—Cu1—Cu1^i^	83.73 (5)	O4—C2—O3	127.3 (2)
O2^i^—Cu1—Cu1^i^	81.54 (5)	N2—C3—N1	112.5 (2)
O4^i^—Cu1—Cu1^i^	83.64 (5)	N3—C4—N1	112.4 (2)
N1—Cu1—Cu1^i^	175.78 (5)	N1—C5—N1^ii^	111.5 (3)
H3A—C3—N1	109.11	C3—N2—C3^ii^	107.9 (3)
H3B—C3—N1	109.13	C3—N2—C6	108.8 (2)
H3A—C3—N2	109.08	H6A—C6—N2	109.20
H4B—C4—N3	109.06	H6B—C6—N2	109.20
C4—N1—C5	107.9 (2)	H6A—C6—N3	109.18
C4—N1—C3	107.8 (2)	H6B—C6—N3	109.18
H4A—C4—N1	109.08	C3^ii^—N2—C6	108.8 (2)
H4B—C4—N1	109.13	C4^ii^—N3—C4	107.6 (3)
C5—N1—C3	107.6 (2)	C4^ii^—N3—C6	108.5 (2)
H5A—C5—N1	109.35	C4—N3—C6	108.5 (2)

Symmetry codes: (i) −*x*, −*y*+1, −*z*; (ii) *x*, −*y*+3/2, *z*.

## References

[bb1] Bruker (1997). *SMART* and *SAINT* Bruker AXS Inc., Madison, Wisconsin, USA.

[bb2] Chiari, B., Piovesana, O., Tarantelli, T. & Zanazzi, P. F. (1988). *Inorg. Chem.* **27**, 3246–3248.

[bb3] Dreyfors, J. M., Jones, S. B. & Sayed, Y. (1989). *Am. Ind. Hyg. Assoc. J.* **50**, 579–585. 10.1080/15298668991375191 2688388

[bb4] Kirillov, A. M. (2011). *Coord. Chem. Rev.* **255**, 1603–1622.

[bb5] Konar, S., Mukherjee, P. S. M., Drew, G. B., Ribas, J. & Chaudhuri, N. R. (2003). *Inorg. Chem.* **42**, 2545–2552. 10.1021/ic020549u 12691560

[bb6] Pickardt, J. (1981). *Acta Cryst.* B**37**, 1753–1756.

[bb7] Sheldrick, G. M. (1996). *SADABS* University of Göttingen, Germany.

[bb8] Sheldrick, G. M. (2008). *Acta Cryst.* A**64**, 112–122. 10.1107/S0108767307043930 18156677

[bb9] Sun, C. Y., Liu, S. X., Liang, D. D., Shao, K. Z., Ren, Y. H. & Su, Z. M. (2009). *J. Am. Chem. Soc.* **131**, 1883–1888. 10.1021/ja807357r 19146450

[bb10] Wang, S., Hu, M.-L. & Ng, S. W. (2002). *Acta Cryst.* E**58**, m242–m244.

[bb11] Wu, B. & Wang, G. (2004). *Acta Cryst.* E**60**, m1764–m1765.

